# Rapidly destructive hip disease causative factors: A case report and literature review

**DOI:** 10.1097/MD.0000000000046625

**Published:** 2025-12-19

**Authors:** Hao Mei, Hang Su, Yubao Yang, Jie Mei, Yunxin Sun, Qiang Shang, Luchun Sun, Jinqing Kan, Xiaobing Chen, Peilei Sun

**Affiliations:** a Department of Orthopedics, Linyi Maternal and Child Health Hospital (graduate school campus), Linyi City, Shandong Province, China; b Department of Orthopedics, Weihai Municipal Hospital, Cheeloo College of Medicine, Shandong University, Weihai City, Shandong Province, China; c Shandong Medical College, Linyi City, Shandong Province, China; d Department of Rehabilitation Medicine, Linyi Maternal and Child Health Hospital (graduate school campus), Linyi City, Shandong Province, China.

**Keywords:** osteolysis, rapidly destructive hip disease, syphilitic bone disease, total hip arthroplasty, total knee arthroplasty

## Abstract

**Rationale:**

It is a rare disease with an unknown etiology and unclear mechanisms, characterized by rapid progression and severe bone destruction, which often leads to late clinical diagnosis and treatment. This study reviewed the literature related to cases of rapid destructive hip disease and the potential causative factors of the disease to offer insights for further diagnosis and treatment.

**Patients concerns:**

A 45-year-old female patient with a history of syphilis initially underwent a total knee arthroplasty due to ineffective conservative management of degenerative left knee disease. Eight months after surgery, the patient developed pain and limited mobility in the ipsilateral hip.

**Diagnoses:**

Imaging examinations, including radiography, 3-dimensional computed tomography (3D CT), and nuclear magnetic resonance imaging, revealed osteolytic destruction of the femoral head and neck.

**Interventions:**

This condition was initially suspected to be a rapidly destructive hip disease, which was treated with left total hip arthroplasty, and was confirmed to be a rapidly destructive hip disease by postoperative pathology.

**Outcomes:**

Postoperative pathology confirmed a diagnosis of rapidly destructive hip disease. The patient recovered well after the surgery, demonstrating good function in both hip and knee joint activities. However, at a later follow-up visit, it was learned that the patient had 4 posterior dislocations. Upon closer examination, the causes were all due to unintentional postures or maneuvers that induced the dislocations.

**Lessons:**

When rapid destructive hip disease is suspected, prompt and thorough evaluation is required. Once diagnosed, total hip arthroplasty should be performed as soon as possible to minimize the risk of postoperative complications such as dislocation, loosening, and infection. In the postoperative period, you should avoid movements that may cause dislocation, such as bending over, crossing your legs, sitting on a low stool, etc.

## 1. Introduction

The origin and development of rapidly destructive hip disease (RDHD) remains largely unknown. Initially introduced by Forestier in 1957 and later termed by Liquesce^[1]^ and Postel^[2]^ in 1970 as “rapidly destructive hip disease,” it included a uniform description of chondrolysis exceeding 2 mm within a year or joint space constriction surpassing 50% in a year, in the absence of other types of swiftly damaging arthropathy. This condition is marked by swift, advancing deterioration of the bone in the femoral head and acetabulum, continuing for weeks or months until the femoral head is entirely removed. In medical settings, it is commonly incorrectly identified as a condition such as rheumatoid arthritis, neuropathic arthropathy, infectious arthritis, femoral head necrosis, seronegative arthritis, or osteoarthritis. Owing to its infrequency, the literature on this topic is scarce. This study involved a retrospective examination of a patient’s case involving rapid destructive hip disease and associated studies, with the aim of enhancing medical professionals’ comprehension, diagnosis, and management of the condition.

## 2. Case presentation

**Figure 1.**
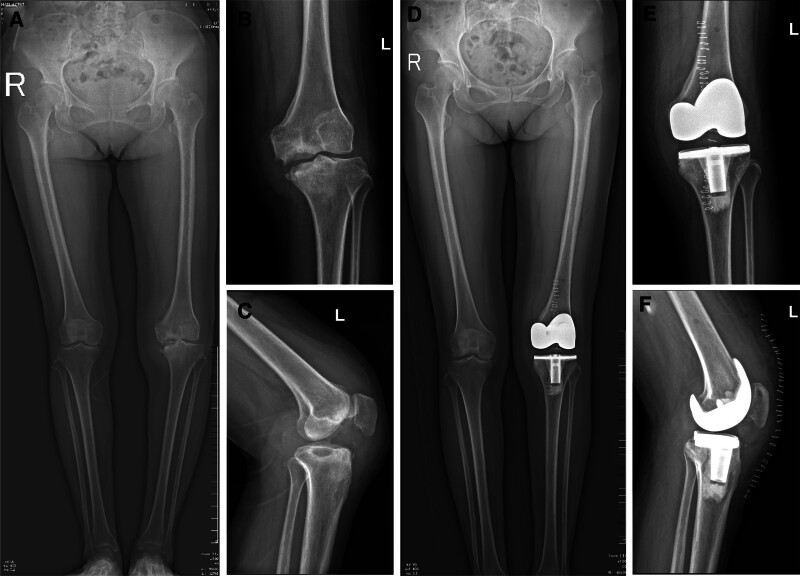
(A–F) Radiographic progression of the left knee: (A) Preoperative full-length bilateral lower extremity radiograph. (B) Preoperative anteroposterior projection radiograph demonstrating severe osteoarthritis, there were different degrees of bone cumbersomening at the edges of the articular surfaces with joint space narrowing. (C) Preoperative lateral projection radiograph. (D) Postoperative full-length bilateral lower extremity radiograph. (E) Postoperative anteroposterior projection radiograph demonstrating the artificial prosthesis was in a good position without loosening or fracture. (F) Postoperative lateral projection radiograph.

**Figure 2.**
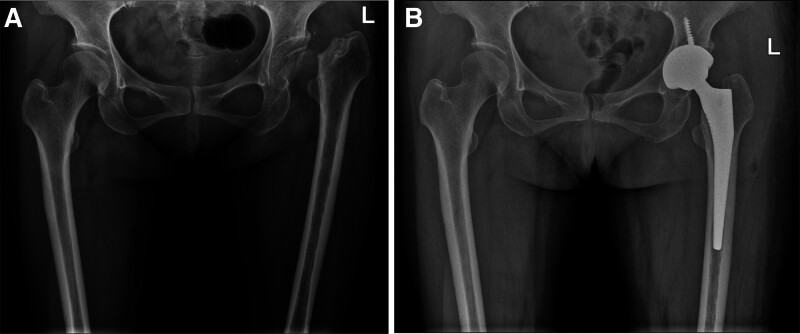
(A–B) Radiographic progression of the left hip: (A) Preoperative anteroposterior projection radiograph demonstrating disappearance of femoral head dissolution. (B) Postoperative anteroposterior projection radiograph demonstrating the artificial prosthesis was in a good position without loosening or fracture.

**Figure 3.**
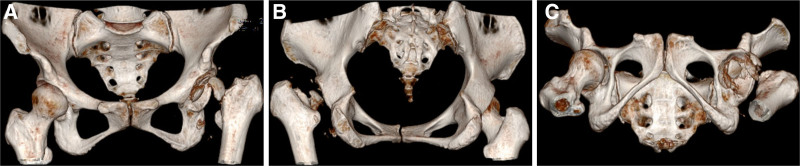
(A–C) Preoperative 3-dimensional computed tomography examination of the hip: (A) Preoperative anteroposterior 3-dimensional computed tomography demonstrating the normal structure of the left femoral head and femoral neck disappears, and multiple irregular bone fragments can be seen, with rounded edges; the bone density of the left acetabulum decreases, and the local bone structure is irregular, and the left femur is shifted to the outside and the top. (B) Preoperative postero-anterior 3-dimensional computed tomography. (C) Preoperative bottom 3-dimensional computed tomography.

**Figure 4.**
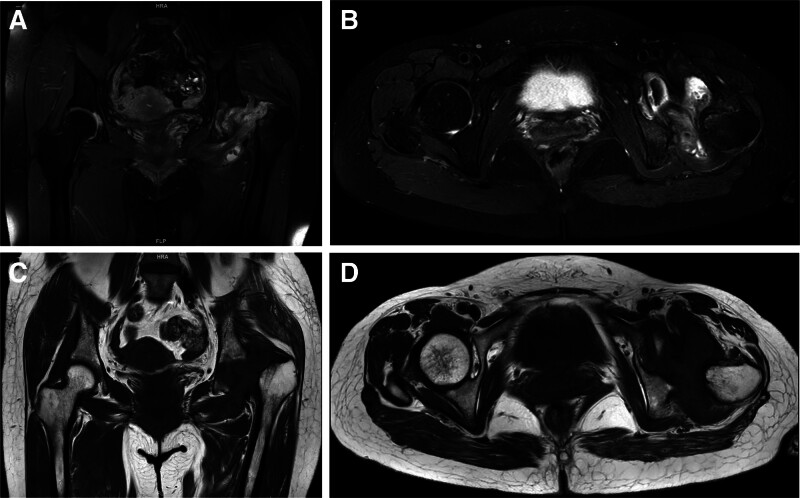
(A–D) Preoperative nuclear magnetic resonance imaging of the hip: (A) Preoperative anteroposterior nuclear magnetic resonance imaging demonstrating the normal structure of the left femoral head and femoral neck disappeared, and a small residual bony signal shadow could be seen. (B) Preoperative horizontal nuclear magnetic resonance imaging demonstrating the corresponding part of the synovial membrane was thickened, the bursa was swollen, and showed flaky FS-PDWI high signal, and a small liquid signal could be seen in the joint space. (C) Preoperative anteroposterior nuclear magnetic resonance imaging demonstrating the corresponding part of the left femur was shifted outwardly and superiorly. (D) Preoperative horizontal nuclear magnetic resonance imaging demonstrating the adjacent femoral end and acetabulum could be seen with flaky FS-TPD2WI high signal.

**Figure 5.**
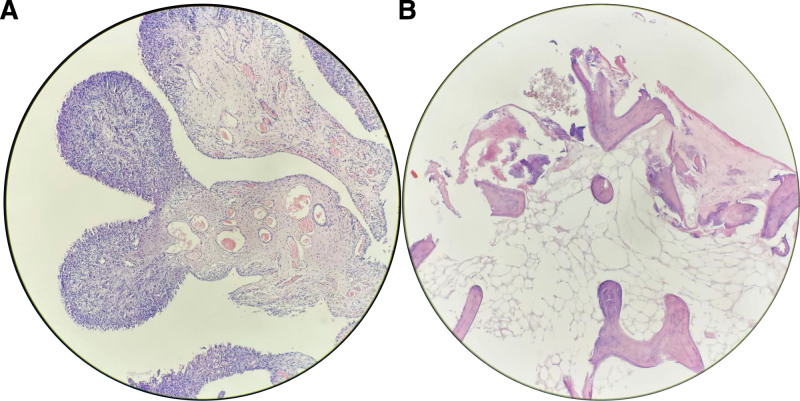
(A–B) Hematoxylin-eosin staining of synovial, residual femoral head tissue (×10). (A) The synovial membrane showed localized papillary hyperplasia with vasodilatation, congestion, and chronic inflammatory cell infiltration. (B) The bone and cartilage showed localized osteolysis with focal bone destruction.

A 45-year-old female presented to our hospital with left knee pain and limited mobility for 1 year. She had no history of infectious diseases prior to admission. Knee range of motion was 0-0-90°. Diagnosis was left knee osteoarthritis (OA). Preoperative serological screening for infectious diseases revealed positive syphilis results: positive syphilis TRUST semi-quantitative test and positive syphilis TPPA qualitative test. Left knee radiographs (Fig. 1A–C). Kellgren–Lawrence (K–L) grade IV. Under intra-articular anesthesia, total left knee surface arthroplasty was performed. Postoperative radiographs demonstrate normal alignment of both lower limbs. (Fig. 1D–F). At 2 weeks postoperatively, the sutures were removed, and the range of motion was 0-0-100°. One month postoperatively, the range of motion was 0-0-105°. Two months postoperatively, the range of motion was 0-0-110°. Eight months after knee surgery, the patient presented with left hip pain and restricted movement. No trauma (e.g., fall, car accident) occurred during this period. The patient intermittently took oral nonsteroidal anti-inflammatory drugs at a local hospital and received intramuscular injections of benzathine penicillin (2.4 million units) for syphilis treatment. Physical examination revealed no significant swelling over the left knee joint, with a range of motion of 0-0-110°. Mild soft tissue swelling was noted over the left hip joint with deep tenderness anteriorly and posteriorly over the buttock. The hip joint range of motion was limited. Squatting was painful and restricted in the left hip. Pain was elicited with hip internal and external rotation. The 4-word test was positive. Harris score was 40 points. A radiograph taken 8 months after left knee arthroplasty showed an intact left femoral head (Fig. 1D–F). Eight months later, hip radiograph (Fig. 2A), 3-dimensional computed tomography (CT) scan (Fig. 3), and magnetic resonance imaging (MRI) (Fig. 4) revealed the disappearance of the left femoral head and femoral neck. Based on the preoperative physical examination and imaging studies, a diagnosis of rapidly progressive hip osteoarthritis was confirmed. Total hip arthroplasty was performed under combined spinal-epidural anesthesia. Intraoperative exploration revealed the absence of the left femoral head with near-complete dissolution. After prosthesis placement and reduction, the joint demonstrated stability and a good range of motion. Synovial tissue and residual femoral head material were obtained intraoperatively and stained with hematoxylin and eosin (Fig. 5). Postoperative bilateral hip radiographs (Fig. 2B) demonstrated a well-positioned prosthetic femoral head with no signs of loosening or fracture. During nearly 3 years of follow-up, the patient experienced 4 episodes of hip dislocation. Detailed history taking revealed no specific actions or postures that the patient recalled causing the dislocations.

## 3. Discussion

**Figure 6.**
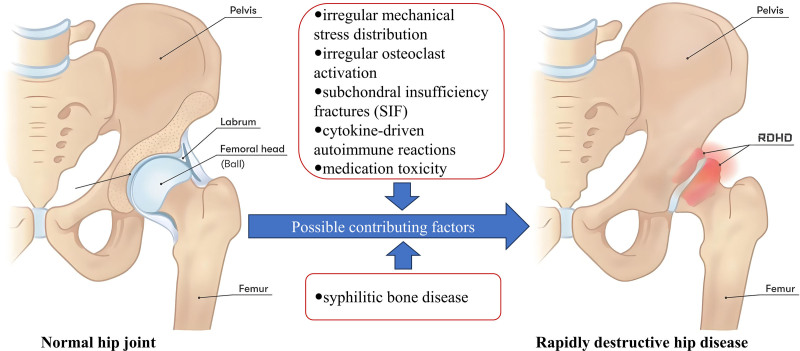
Possible causative factors for rapidly destructive hip disease.

RDHD lesions rapidly advance, characterized by osteolysis and damage to the femoral head and acetabulum in weeks to months, making the disease’s length a key diagnostic aspect, ranging from initial pain to osteolysis and resorption in less than a year. Research has indicated that initial hip pain detected through hip joint radiograph is typical, but within a mere 6 weeks of heightened pain and functional constraints, these radiograph reveal bone degradation and absorption in the femoral head’s subchondral bone, along with collapse and flattening.^[3]^ This illness mainly affects elderly women, affecting both sides in approximately 80% to 90% of cases.^[4]^ Approximately 1% to 5% of patients undergoing total hip arthroplasty exhibit RDHD.^[5–7]^ The origin of RDHD remains a mystery, with numerous academics tributing it to various elements and proposing several theories, primarily centered on some key aspects (Fig. 6): irregular mechanical stress distribution, irregular osteoclast activation, subchondral insufficiency fractures (SIF), cytokine-driven autoimmune reactions, and medication toxicity.

OA onset is primarily attributed to mechanical and cartilage deterioration. Research indicates a reduction in the contact area between the acetabulum and femoral head coupled with heightened biological stress on the residual contact surface in RDHD patients, suggesting a link between RDHD and the modified distribution of biological stress.^[8]^ Nuclear magnetic resonance imaging scans of RDHD patients showed posterior pelvic tilt and acetabular labral reflection in the impacted hip, which is believed to facilitate labral absorption into the joint cavity, leading to labral reflection upon hip flexion.^[9]^ Likewise, research indicates that severely deteriorated and fragile labral rim cartilage tends to detach into the joint cavity; however, well-preserved labral rim cartilage leads to stress accumulation at the reflection point, increasing the risk of SIF in the acetabulum and femoral head.^[10]^ Consequently, changes in pelvic stress due to mechanical elements such as rearward pelvic tilt and labral reflection may initially contribute to swift joint damage. In this study, the patient underwent complete arthroplasty of the left knee due to degeneration, followed by radiograph after surgery (Fig. 1B), which revealed no alteration in the force line of the left lower limb.

This hypothesis posits a connection between RDHD and SIF, attributed to their analogous imaging manifestations. Multiple round-shaped granulomas in the central bone marrow are encircled by shapeless pieces, bone marrow pieces, and articular cartilage tissues encircled by histiocytes and osteoclasts, suggesting the likelihood of swift joint damage when SIF is combined with osteoporosis in older women.^[11,12]^ Furthermore, nuclear magnetic resonance imaging scans revealed a low-density band beneath the cartilage in the weight-bearing region of the femoral head in patients with RDHD, indicating significant involvement of SIF in RDHD development.^[13]^ The patients nuclear magnetic resonance imaging in this study showed no symptoms similar to those of SIF. However, RDHD has been proposed to manifest through a distinct mechanism.

Unusual stimulation of osteoclasts. Research has indicated the presence of macrophage-abundant granulomas in the bone marrow and synovium, along with unusual osteoclast activation in the synovial fluid of patients with RDHD. It is theorized that the increased osteoclastic activity triggered by this unidentified factor interferes with the osteogenic-osteoclastic balance, playing a key role in the swift deterioration of bone on the femoral head and acetabular side.^[14]^ Research has also indicated the presence of osteoclasts in the synovial fluid of individuals with RDHD, and it is theorized that RDHD’s emergence of RDHD is linked to the unusual activation of osteoclasts in the joint fluid.^[15]^ Research also indicates that RDHD rapidly breaks down bone, likely due to an increase in matrix metalloproteinase 3.^[16]^ Our research revealed an increase in synovial hyperplasia and an accumulation of synovial fluid during surgery, with hematoxylin-eosin staining verifying osteolytic destruction (Fig. 5), regrettably, there was no additional confirmation of unusual osteoclast activation or a rise in matrix metalloproteinase 3 levels.

Some scholars have proposed a link between intra-articular injections and the emergence of RDHD. Intra-articular injections, such as sodium vibrate, steroids, and local anesthetics, are considered economical methods for alleviating pain during the initial or severe stages of hip arthropathy. Research indicates that older patients undergoing intra-articular hormonal injections for primary OA experience more intense symptoms and a greater likelihood of developing RDHD.^[17]^ Research indicates that hormone injections within joints evolve into RDHD within 2 months, characterized by a gradual emergence and swift deterioration of joints.^[18]^ Furthermore, the use of local anesthetics has been linked to cartilage deterioration. Study has revealed a correlation between the strength of the impact of the local anesthetic and the timing and dosage of its administration^[19]^; thus, it is crucial to consider the possible harmful consequences of steroids and local anesthetics in intra-articular injections on the articular cartilage.

Various academics have suggested alternative origins for RDHD, including conditions such as syphilitic bone disease, where bone involvement is a long-term phenomenon, the extent of which correlates with the length of syphilis, typically manifesting in the second and third phases of syphilis, and disease duration ranging from 2 to 4 years or longer, termed syphilitic bone disease, marked by its prolonged nature and mild symptoms. In the initial stages of syphilis, the likelihood of destructive bone lesions is considerably lower, as research indicates a mere 0.15% occurrence rate of such conditions in early syphilis cases.^[20]^ In this study, the patient was hospitalized at an 8-month interval, contradicting the usual pattern of syphilitic bone disease.

## 4. Conclusions

Given the infrequency of RDHD in medical settings and its prevalence in advanced disease stages, continuous and thorough observation and research on its development is challenging. The exact process remains a mystery, with varying opinions among researchers, possibly because of the combined impact of several elements. Beyond thorough assessments, it is crucial to guide patients suspected of having RDHD to consistently check in and receive arthroplasty post-diagnosis to prevent severe joint damage, substantial bone mass loss, and other issues, such as heightened trauma, loosening, dislocation, and infection.

## Author contributions

**Data curation:** Yunxin Sun, Qiang Shang.

**Investigation:** Jie Mei.

**Supervision:** Yubao Yang, Jinqing Kan, Xiaobing Chen, Peilei Sun.

**Visualization:** Luchun Sun.

**Writing – original draft:** Hao Mei.

**Writing – review & editing:** Hang Su.
